# Effects of reactive oxygen species and neutrophils on endothelium-dependent relaxation of rat thoracic aorta

**DOI:** 10.2478/v10102-011-0029-3

**Published:** 2011-12

**Authors:** Viktor Bauer, Ružena Sotníková, Katarína Drábiková

**Affiliations:** Institute of Experimental Pharmacology & Toxicology, Slovak Academy of Sciences, SK-84104 Bratislava, Slovakia

**Keywords:** ROS, neutrophils, aorta

## Abstract

Reactive oxygen species (ROS) are produced in different metabolic processes including the respiratory burst of neutrophils accompanying local inflammation. The aim of this study was to analyze the effects of N-formyl-methionyl-leucyl-phenylalanine (FMLP)-activated neutrophils, isolated from the guinea pig peritoneal cavity, on isolated rings of a large (conduit) artery, the rat thoracic aorta. FMLP-activated neutrophils enhanced the basal tension increased by α_1_-adrenergic stimulation. In phenylephrine-precontracted aortae, they elicited marked contraction, while in noradrenaline-precontracted rat aortal rings they caused a biphasic response (contraction-relaxation). To eliminate interaction of activated neutrophils with catecholamines, in the subsequent experiments the basal tension was increased by KCl-induced depolarization. Activated neutrophils evoked a low-amplitude biphasic response (relaxation-contraction) on the KCl-induced contraction. Not only the acetylcholine- and A_23187_-induced relaxations but also the catalase sensitive hydrogen peroxide (H_2_O_2_) elicited contractions were endothelium-dependent. Even though the acetylcholine-induced relaxation was changed by activated neutrophils and by the ROS studied, their effects differed significantly, yet none of them did eliminate fully the endothelium-dependent acetylcholine relaxation. The effect of activated neutrophils resembled the effect of superoxide anion radical (O_2_^•–^) produced by xanthine/xanthine oxidase (X/XO) and differed from the inhibitory effects of Fe_2_SO_4_/H_2_O_2_-produced hydroxyl radical (^•^OH) and H_2_O_2_. Thus O_2_^•–^ produced either by activated neutrophils or X/XO affected much less the endothelium-dependent acetylcholine-activated relaxation mechanisms than did ^•^OH and H_2_O_2_. In the large (conduit) artery, the effects of activated neutrophils and various ROS (O_2_^•–^, ^•^OH and H_2_O_2_) seem to be more dependent on muscle tension than on endothelial mechanisms.

## Introduction

Reactive oxygen species (ROS) damage different tissues including those of the cardiovascular system. Maugeri *et al*. (2009) have suggested that processes which activate macrophages in the vessel wall are responsible for systemic manifestations and for local vessel injury. Local inflammation and respiratory burst of polymorphonuclear neutrophils (PMNs) results in progression of various cardiovascular diseases. ROS generated in respiratory burst of neutrophils may remain inside the cells or be released from them to the extracellular space (Karlsson and Dahlgren, 2002; Nosál *et al.*, 2009) and diffuse to the vessel wall. Vascular endothelial cells are the first targets of PMNs and were found to be key participants in the development of the altered resistance vessel reactivity (Mullane and Pinto, 1987; Csaki *et al*., 1991; Akopov *et al*., 1992; Tsao *et al*., 1992; De Kimpe *et al*., 1993) in ischemia-induced (Tsao and Lefer, 1992; Kadletz *et al*., 1994; Pétrault *et al*., 2005) and inflammation-mediated (Murphy *et al*., 1998) cardiovascular injuries.

The response of neutrophils to several biologically active substances, *e.g.* to arachidonic acid, phorbol myristate acetate (PMA), N-formyl*-*methionyl-leucyl-phenylalanine (FMLP) or to the calcium ionophore A_23187_, mimics the inflammation-induced burst of neutrophils as well as the production and release of superoxide anion radical (O_2_^•–^). Subsequently, O_2_^•–^ may initiate formation of derived ROS: hydrogen peroxide (H_2_O_2_), singlet oxygen, hydroxyl radical (^•^OH) and hypochlorous acid (Sand *et al*., 2003a, Bauer *et al*., 2008; Jančinová *et al.,* 2011).

The aim of the present study was therefore to compare the effects of FMLP-activated isolated peritoneal neutrophils with those of different exogenously applied ROS (O_2_^•–^, H_2_O_2_ and ^•^OH) on endothelium-dependent acetylcholine-induced relaxation of a large (conduit) artery.

## Methods

The experimental procedures were conform to the Guide for the Care and Use of Laboratory Animals (National Institutes of Health and State Veterinary and Food Product Inspection of the Slovak Republic) and were approved by the Animal Care and Use Committee at the Institute of Experimental Pharmacology and Toxicology of the SASc and by the State Veterinary and Food Product Administration of the Slovak Republic.

The animals used (guinea-pigs and rats) were killed by cervical dislocation and exsanguination.

### Chemicals

Calcium ionophore (A_23187_), acetylcholine (Ach), glycogen, N-formyl-L-methionyl-L-leucyl-L-phenylalanine (FMLP), percoll, phenylephrine, sodium citrate tribasic dehydrate, xanthine (X), xanthine oxidase (XO), *N*_ω_-Nitro-L-arginine methyl ester hydrochloride (L-NAME) were all from Sigma and Sigma-Aldrich Chemie (Germany), trypan blue from Fluka (Switzerland), FeSO_4_, H_2_O_2_ and the other chemicals were of p.a. purity purchased from Lachema Brno (Czech Republic).

### Solutions

Phosphate buffer saline A (PBA) contained (in mmol/l): NaCl 136, KCl 2.6, Na_2_HPO_4_ 8.0, KH_2_PO_4_ 1.5, pH 7.4; phosphate buffer saline B (PBB): NaCl 136, KCl 2.6, Na_2_HPO_4_ 8.0, KH_2_PO_4_ 1.5, CaCl_2_ 0.6, MgCl_2_ 0.5 and glucose 5.6, pH 7.4; and the physiological salt solution (PSS): NaCl 112.0, KCl 5.0, KH_2_PO_4_ 1.0, MgSO_4_ 1.2, CaCl_2_ 2.5, NaHCO_3_ 25.0 and glucose 11, pH 7.4. The high potassium depolarizing salt solution (DSS) was the same as PSS with the only difference that the concentration of KCl was increased with equimolar reduction of NaCl concentration to achieve 100 mmol/l of KCl.

### Isolation and preparation of neutrophils

Neutrophils were isolated from the peritoneal exudate of male Trick guinea-pigs (450–600 g body weight) as described recently (Bauer *et al*., 2008; 2011). The animals were sacrificed 14 to 16 h after intraperitoneal injection of 20 ml of 1.2% glycogen in 0.9 % NaCl. The abdomen was gently massaged after injection of 20 ml of 0.4% trisodium citrate in 0.867% NaCl into the peritoneal cavity and the peritoneal exudate was collected and filtrated. All the following procedures were performed at 4 °C. Cell suspension of the peritoneal exudate were washed with PBA and centrifuged for 90 s at 2 500 rpm. Erythrocytes were removed by hypotonic treatment. The neutrophil pellets were resuspended in PBA and 2 ml of their suspension was layered on the discontinuous density gradient of Percoll (1.5 ml of 1.095 g/ml and 1.5 ml of 1.077 g/ml). After centrifugation on the Percoll density gradient for 10 min at 2500 rpm, neutrophils were collected from the interface and washed with PBA and centrifuged two times for 90 s at 2000 rpm. The neutrophils resuspended in PBA were counted using Coulter Counter Electronics (England), their viability was assessed using trypan blue and the activity was confirmed also by the ability of ROS production (Sugioka *et al*., 1986; Nosál *et al*., 2002; Bauer *et al.*, 2011).

Neutrophils stored in *PBA* stock solutions at 4°C were used for experiments within the following 2–3 hours.

### Isolation and preparation of rat thoracic aorta

The thoracic aorta removed from male Wistar rats (250–300 g body weight) was immersed in PSS. Adherent tissues were removed and 2 mm long rings were cut. Care was taken not to damage the endothelium.

**Figure 1:**
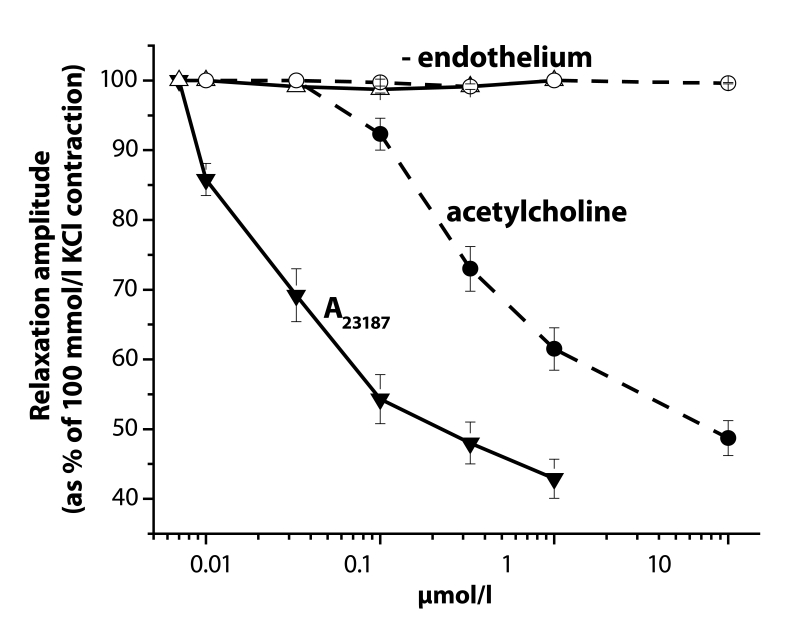
Reduction of acetylcholine- and A_23187_-induced relaxation of 100 mmol/l KCl-precontracted rings of the rat thoracic aorta by endothelium removal. Closed symbols – in the presence of endothelium, open symbols in its absence.

The rings were mounted between two platinum hooks. The tissue chamber contained PSS, bubbled with a mixture of 95% O_2_ and 5% CO_2_ (pH=7.4) at 37 °C (Sotníková *et al*., 1994). The rings were stretched passively to the resting tension of 20 mN and were allowed to equilibrate for 1 hr. During this period of time, PSS was repeatedly replaced and the tension was readjusted to 20 mN. The isometric tension was recorded using a strain gauge transducer (Experimentria, Hungary) coupled to a potentiometric pen recorder (Metrimpex, Hungary). In part of the experiments, the endothelium was destroyed by gentle rubbing of the lumen of the preparations with a forceps. The presence of a functional vascular endothelium was confirmed by the ability of acetylcholine (0.01–10 µmol/l) and of the calcium ionophore A_23187_ (0.01–1 µmol/l) to produce vascular relaxation at the plateau of the 100 mmol/l KCl-induced contraction (Figure 1).

H_2_O_2_ (0.01–1 mmol/l) was added as pure chemical, O_2_^•–^ was generated by xanthine (X, 0.1 mmol/l) with xanthine oxidase (XO, 0.1 IU/ml), and ^•^OH was produced by FeSO_4_ (0.1 mmol/l) with H_2_O_2_ (0.15 mmol/l).

### Analysis of data

All values are given as mean ± SEM of at least 5–7 experiments. The statistical significance of differences between means was established by Student's *t*-test and the values of *p*<0.05 were considered statistically significant.

The mechanical responses are expressed generally as percentages of the 100 mmol/l KCl DSS-induced contraction measured at the beginning of each experiment.

## Results

### Effects of neutrophils on phenylephrine-and noradrenaline-precontracted aortae

**Figure 2:**
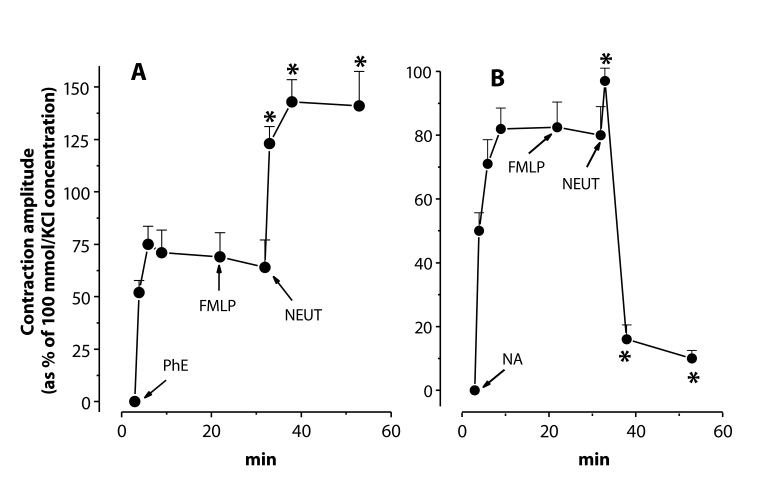
Responses of phenylephrine (**A** – PhE, 1 µmol/l)- and noradrenaline (**B** – NA, 1 µmol/l)- precontracted rings of the rat thoracic aorta to N-formyl*-*methionyl-leucyl-phenylalanine (FMLP, 0.1 µmol/l) and to neutrophils (NEUT, 10^6^ cells/ml) in the presence of FMLP in the bathing fluid. * – significant differences from control (*p<*0.01).

To study the effects of activated neutrophils, the concentration of 1 µmol/l was selected from various concentrations (0.1–10 µmol/l) of phenylephrine or noradrenaline. Entry of adrenergic receptor stimulants in the given concentration to the bathing fluid induced sustained contractions with the amplitude of about 70–85% of those evoked by 100 mmol/l KCl. Native neutrophils (10^6^ cells/ml final concentration in the chamber) similarly as FMLP (0.1 mol/l) alone had no significant effect on the phenylephrine- or noradrenaline-elevated tension. In the presence of FMLP, however, neutrophils markedly and homogenously elevated the phenylephrine-increased basal tension of the rat thoracic aortic rings. The phenylephrine-evoked tension increase was doubled by the activated neutrophils (Figure 2A). In contrast, on noradrenaline-pretreated aortae, activated neutrophils elicited a biphasic response (contraction-relaxation). The initial transient contraction did not reach more than 15–20% of that evoked by 100 mmol/l KCl. In the second relaxation phase, the noradrenaline-induced precontraction was almost completely eliminated (Figure 2B).

### Endothelium-dependent relaxation

**Figure 3:**
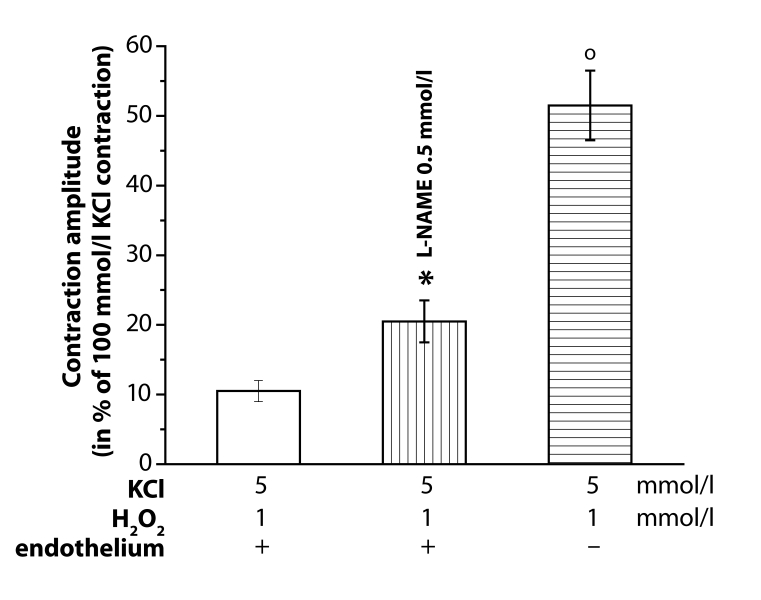
Increase of the H_2_O_2_ (1 mmol/l)-induced contraction amplitude of resting (KCl, 5 mmol/l) rings of the rat thoracic aorta induced by L-NAME (0.5 mmol/l) or endothelium removal (–). Responses in the presence of endothelium (+). Significant differences from control are: *p<0.05; ^o^*p<*0.01.

To prove the possible ROS-induced effects on the acetylcholine (Ach, 10 µmol/l)-evoked endothelium-dependent relaxation of 100 mmol/l KCl- precontracted aortae, H_2_O_2_ pretreatment was used. In the presence of endothelium, H_2_O_2_ (0.01, 0.1 and 1 mmol/l) contracted the aortal rings. The evoked contraction amplitude was significantly increased both by 0.5 mmol/l L-NAME-pretreatment and the endothelium removal (Figure 3).

**Figure 4:**
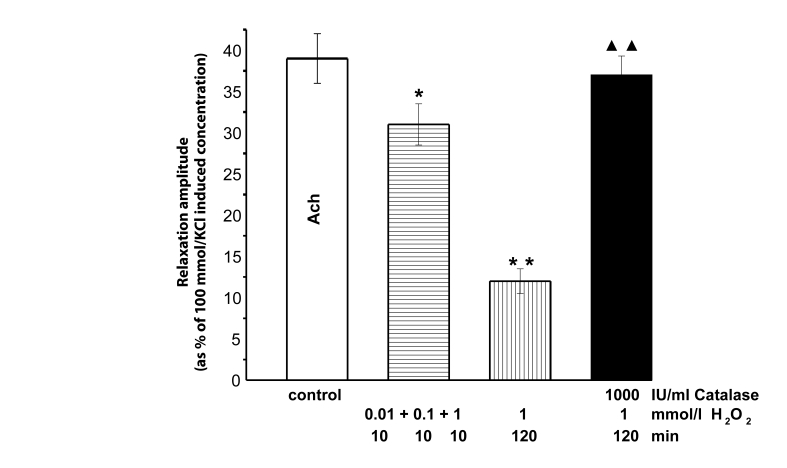
Decrease of acetylcholine (Ach, 10 µmol/l)-induced relaxation amplitude of KCl (100 mmol/l)-precontracted rings of the rat thoracic aorta. Before (control) and in the presence of cumulative(0.01, 0.1 and 1 mmol/l) H_2_O_2_ application (in 10min intervals) as well as in the 120 min-lasting presence of 1 mmol/l H_2_O_2_ without and during catalase (1000 IU/ml) treatment. Significant differences from control are: *p<0.05; **p<0.01, between catalase treated and untreated aortae ▴▴*p<*0.01.

Cumulative application of H_2_O_2_ (0.01, 0.1 and 1 mmol/l) in 10 min intervals attenuated the acetylcholine-produced relaxation by 10–20%. This reduction was ameliorated by 120 min-lasting pretreatment with H_2_O_2_ (1 mmol/l). In the presence of catalase (1000 IU/ml), the effects of H_2_O_2_ were absent even in its highest concentration (1 mmol/l) (Figure 4).

### Effects of neutrophils on potassium chloride-precontracted aortae

**Figure 5:**
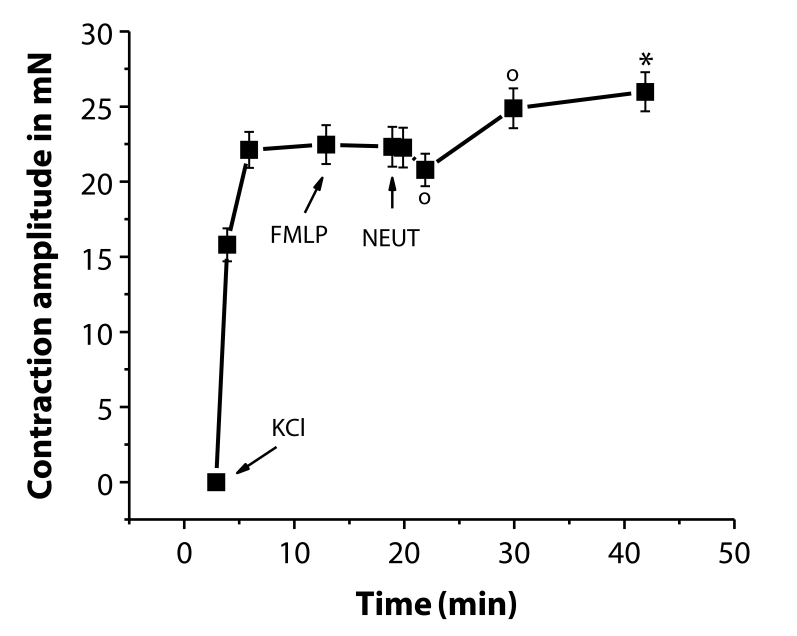
Biphasic responses of KCl (100 mmol/l)-precontracted rings of the rat thoracic aorta to N-formyl-methionyl-leucyl-phenylalanine (FMLP, 0.1 µmol/l) and to neutrophils (NEUT, 10^6^ cells/ml) in the presence of FMLP in the bathing fluid. Significant differences are: ^o^*p<*0.05; *p<0.01 from the contraction level before neutrophil administration.

High potassium (100 mmol/l)-containing DSS increased the basal tension of the aortal rings by 20 to 25 mN. FMLP alone did not affect the DSS-elevated tension. Introduction of neutrophils into the FMLP-containing bathing fluid induced a moderate transient initial relaxation followed by elevation of the tension above the KCl-induced level (Figure 5).

### Acetylcholine-induced relaxation in the presence of activated neutrophils or ROS

**Figure 6:**
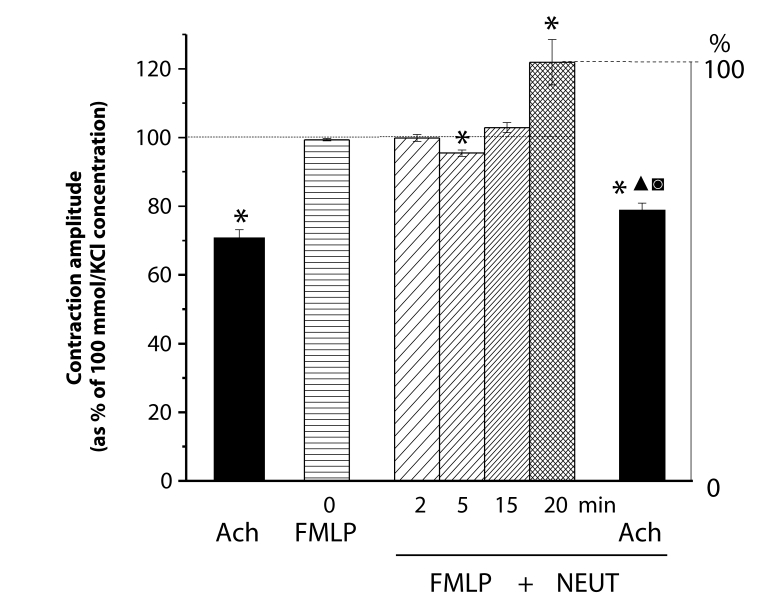
Responses of KCl (100 mmol/l)-precontracted rings of the rat thoracic aorta to acetylcholine (Ach, 10 µmol/l) before and after 20 min-lasting treatment by N-formyl-methionyl-leucyl-phenylalanine (FMLP, 0.1 µmol/l) activated neutrophils (NEUT, 10^6^ cells/ml). Each bar represents the mean ± SEM of 5–7 experiments. Significant differences (at least *p<*0.05) are: (*) from the initial KCl-elevated tension, (▴) between the Ach-reached relaxation levels, (▪) between the Ach-induced relaxation amplitudes.

Under control conditions, acetylcholine (10 µmol/l) reduced the 100 mmol/l KCl-elevated muscle tension by 25–40%. In the presence of FMLP, neutrophils induced an initial relaxation followed by contraction. After 20 min-lasting effect of activated neutrophils, the muscle tension stabilized at about 20% above the KCl-induced contraction level. At this level of enhanced tension, acetylcholine markedly relaxed the aortae. The relaxation amplitude was significantly enlarged but the level of the tension reached differed only slightly from that attained under control conditions (Figure 6).

**Figure 7:**
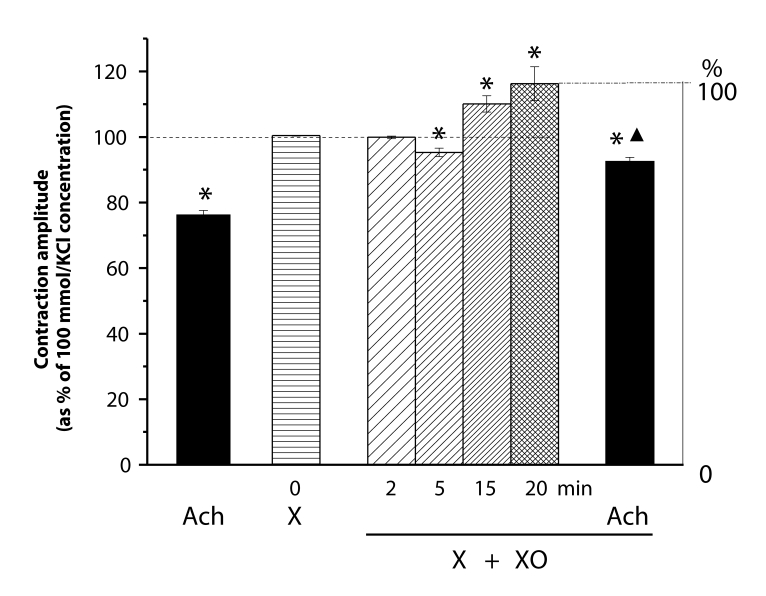
Responses of KCl (100 mmol/l)-precontracted rings of the rat thoracic aorta to acetylcholine (Ach, 10 µmol/l) before and after 20min treatment by xanthine (X, 0.1 mmol/l) and xanthine oxidase (XO, 0.1 IU/ml). Each bar represents the mean ± SEM of 5–7 experiments. Significant differences (at least *p<*0.05) were: (*) from the initial KCl-elevated tension, (▴) between the Ach-reached relaxation levels.

Comparably to FMLP, xanthine (X, 0.1 mmol/l) alone did not affect the KCl-elevated muscle tension. Addition of xanthine oxidase (XO, 0.1 IU/ml) to the xanthine-containing solution caused production of O_2_^•–^, resulting in a similar biphasic response (relaxation-contraction) as described above in the case of activated neutrophils. After 20 min-lasting effect of X/XO, the muscle tension stabilized at about 15% increased tension level. At this level of enhanced tension, acetylcholine relaxed the aortae with almost the same amplitude as under control conditions. The level of the tension reached, however, differed significantly from that in controls (Figure 7).

**Figure 8:**
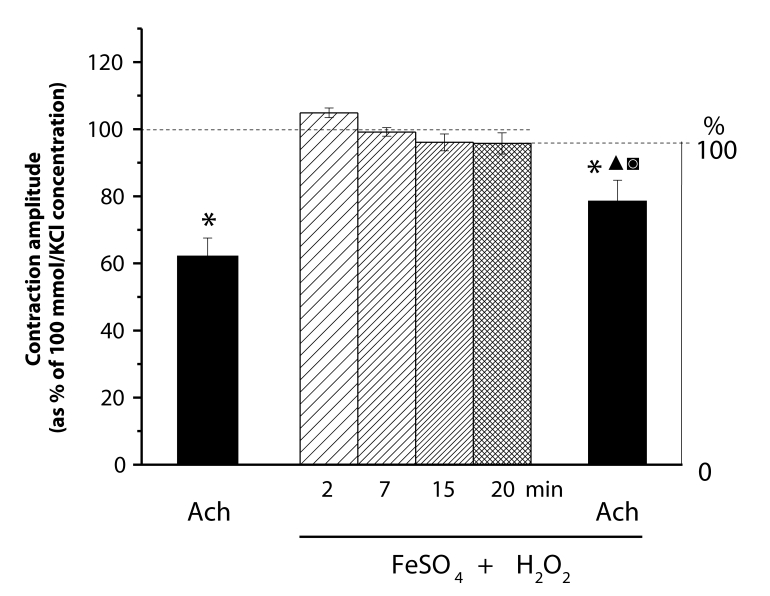
Responses of KCl (100 mmol/l)-precontracted rings of the rat thoracic aorta to acetylcholine (Ach, 10 µmol/l) before and after 20 min treatment by FeSO_4_ (0.1 mmol/l)/H_2_O_2_ (0.15 mmol/l). Each bar represents the mean ± SEM of 5–7 experiments. Significant differences (at least *p<*0.05) are: (*) from the initial KCl-elevated tension, (▴) between the Ach-reached relaxation levels, (▪) between the Ach-induced relaxation amplitudes.

The effects of FeSO_4_ (0.1 mmol)/H_2_O_2_ (0.15 mmol/l)-produced ^•^OH differed from those induced by O_2_^•–^ originated from activated neutrophils and X/XO. The mild alterations of the KCl-elevated muscle tension were not significant. The muscle tension after 20 min-lasting effect of FeSO_4_/H_2_O_2_ was significantly reduced by acetylcholine. The relaxation amplitude and the level of tension reached differed significantly from the values attained under control conditions (Figure 8).

**Figure 9:**
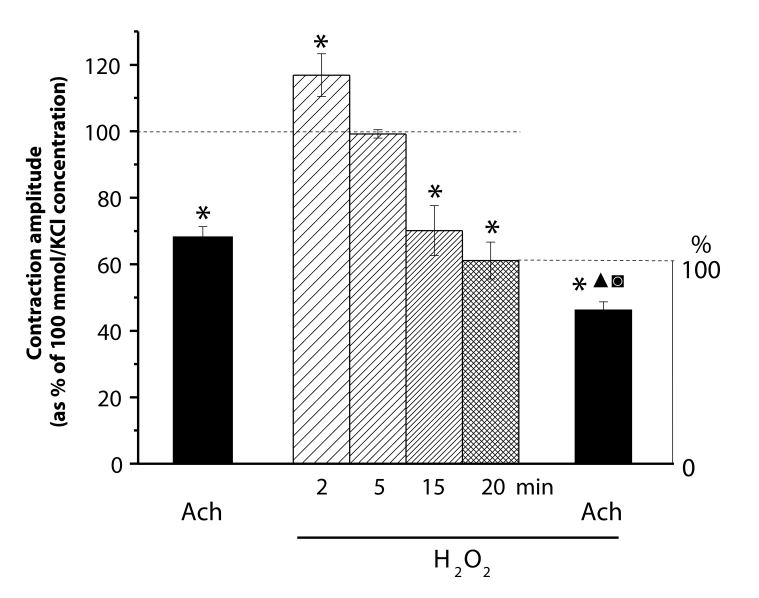
Responses of KCl (100 mmol/l)-precontracted rings of the rat thoracic aorta to acetylcholine (Ach, 10 µmol/l) before and after 20min treatment by H_2_O_2_ (0.1 mmol/l). Each bar represents the mean ± SEM of 5–7 experiments. Significant differences (at least *p<*0.05) are: (*) from the initial KCl-elevated tension, (▴) between the Ach-reached relaxation levels, (▪) between the Ach-induced relaxation amplitudes.

In contrast to the above described effects of activated neutrophils and of X/XO, the KCl-elevated muscle tension was transiently enhanced and then markedly reduced by FeSO_4_/H_2_O_2_ and H_2_O_2_ (1 mmol/l) alone. After 20min lasting action of H_2_O_2_, the reduction was roughly 40%. As found in the experiments with its cumulative and long-lasting action (Figure 2) H_2_O_2_ impaired significantly the acetylcholine-evoked relaxation even in the presence of its high concentration (1 mmol/l) for 20 min in the bathing fluid. The level of the tension reached differed also significantly from that attained under control conditions. The acetylcholine- elicited relaxation amplitude was however smaller than that induced by the other ROS studied (Figure 9).

## Discussion

Unstimulated resting PMNLs release nitric oxide radical (NO^•^) or a similar substance that relaxes vascular tissues (Mehta *et al*., 1991; Chatterjee *et al*., 2007). After adherence to the vascular endothelium, neutrophils migrate to sites of tissue damage, are activated in the inflammatory process and release various vasoactive factors. The contractile effect of the released vasoactive substances may result either from their direct action on the vascular smooth muscle cells or from their indirect effects, *e.g.* due to influence of endothelial cells (Ohlstein and Nichols, 1989). The influence of endothelial cells may cause reduction of the activities or effects of the endothelium-derived autocoids, such as prostacycline, NO or the endothelium-derived hyperpolarizing factor (Ohlstein and Nichols, 1989). As a consequence, the endothelium-dependent vascular relaxation may be affected (Mehta *et al*., 1991). Similarly to the inflammatory process, activation of neutrophils by PMA, FMLP or A_23187_ induces release of contractile factors (Mehta *et al*., 1991), which can be ROS and/or nitrogen species different from NO^•^ (Chatterjee *et al*., 2007). There are different ROS (O_2_^•–^, H_2_O_2_, ^•^OH, singlet oxygen, hypochlorous acid) which can affect smooth muscle tissues (Bauer *et al*., 1999) including vessels and may mimic inflammation-induced responses (Bauer *et al*., 2008; 2011).

By binding to specific membrane receptors on the cell surface, the activated neutrophils trigger intracellular signaling and oxidative burst (McPhail *et al*., 1995). The production of ROS by neutrophils is predominantly derived from the activation of NADPH oxidase in the neutrophil plasma membrane, with concomitant generation of O_2_^•–^ and subsequently derived ROS (H_2_O_2_, ^•^OH, singlet oxygen and hypochlorous acid) (Sand *et al*., 2003a,b). The marked contraction evoked by FMLP-activated neutrophils of aortae precontracted by the α_1_-adrenergic agonist phenylephrine suggests the release of a strongly effective contractile factor. Elimination of the noradrenaline-enhanced muscle tension by the activated neutrophils suggests that the released ROS inactivates noradrenaline probably due to its strong oxidant property. In the experiments analyzing the effects of activated neutrophils, O_2_^•–^, H_2_O_2_ and ^•^OH on the acetylcholine-evoked relaxation, we therefore used KCl instead of α-adrenergic agonists for precontraction. Under such conditions, the characteristics of the effects of activated neutrophils and O_2_^•–^ produced by xanthine/xanthine oxidase differed from those of H_2_O_2_ and ^•^OH produced by FeSO_4_/H_2_O_2_. This suggests that the ROS released from the activated neutrophils is O_2_^•–^ rather than H_2_O_2_ or ^•^OH.

Not all tissues provide the same response to inflammation and the role of vascular endothelial cells in this response is not yet clear. De Kimpe *et al*. (1993) found reduction of the acetylcholine-induced relaxation by PMNs in a resistant vessel (mesenteric artery). They concluded that small amounts of O_2_^•–^ produced by unstimulated PMNs contributed to a decrease in relaxation to acetylcholine by interfering with endothelium-derived NO^•^.

In the aorta, the large (conduit) artery with intact endothelium, none of the ROS used eliminated fully the acetylcholine-induced relaxation. There were, however, significant differences. The ROS effects were at least in part dependent on the induced elevation or reduction of the initial tension. Strong increase of the initial tension by activated neutrophils enlarged the acetylcholine-induced relaxation amplitude. The modest increase of the initial tension by O_2_^•–^ had no significant effect, while the unchanged or reduced initial tension by ^•^OH and H_2_O_2_ attenuated the acetylcholine-evoked relaxation amplitude. In addition to the effects on muscle tension, the effects of ROS on acetylcholine-induced relaxation indicate that the actions of ^•^OH and H_2_O_2_ differ from those of O_2_^•–^, produced either by activated neutrophils or X/XO, also in the endothelium-derived function.

In contrast to the resistant vessels, which regulate organ/area blood supply and distribution, *e.g.* coronary artery (Mullane and Pinto, 1987), middle cerebral artery (Csaki *et al*., 1991; Akopov *et al*., 1992); mesenteric artery (De Kimpe *et al*., 1993) or the whole myocardium (Tsao *et al*., 1992), our results suggest that in large (conduit) arteries like the aorta, the effects of activated neutrophils and various ROS (O_2_^•–^, ^•^OH and H_2_O_2_) are dependent on the evoked muscle tension and their effect on the acetylcholine-induced endothelium-dependent relaxation is probably a secondary phenomenon.
